# The Curious Layperson: Fine-Grained Image Recognition Without Expert Labels

**DOI:** 10.1007/s11263-023-01885-9

**Published:** 2023-09-13

**Authors:** Subhabrata Choudhury, Iro Laina, Christian Rupprecht, Andrea Vedaldi

**Affiliations:** Visual Geometry Group, University of Oxford, Oxford, OX1 3PJ UK

**Keywords:** Fine-grained classification, Clever, Non-expert annotations, Multimodal retrieval

## Abstract

Most of us are not experts in specific fields, such as ornithology. Nonetheless, we do have general image and language understanding capabilities that we use to match what we see to expert resources. This allows us to expand our knowledge and perform novel tasks without ad-hoc external supervision. On the contrary, machines have a much harder time consulting expert-curated knowledge bases unless trained specifically with that knowledge in mind. Thus, in this paper we consider a new problem: fine-grained image recognition without expert annotations, which we address by leveraging the vast knowledge available in web encyclopedias. First, we learn a model to describe the visual appearance of objects using non-expert image descriptions. We then train a fine-grained textual similarity model that matches image descriptions with documents on a sentence-level basis. We evaluate the method on two datasets (CUB-200 and Oxford-102 Flowers) and compare with several strong baselines and the state of the art in cross-modal retrieval. Code is available at: https://github.com/subhc/clever.

## Introduction

**Fig. 1:**
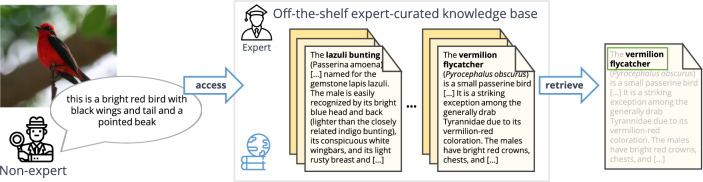
Fine-Grained Image Recognition without Expert Labels. We propose a novel task that enables fine-grained classification without using expert class information (e.g. bird species) during training. We frame the problem as document retrieval from general image descriptions by leveraging existing textual knowledge bases, such as Wikipedia

Deep learning and the availability of large-scale labelled datasets have led to remarkable advances in image recognition tasks, including fine-grained recognition (Wah et al., 2011; Nilsback and Zisserman, 2006; Horn et al., 2017). The problem of fine-grained image recognition amounts to identifying subordinate-level categories, such as different species of birds, dogs or plants. Thus, the supervised learning regime in this case requires annotations provided by domain *experts* or citizen scientists (Van Horn et al., 2015).

While most people, unless professionally trained or enthusiasts, do not have knowledge in such specific domains, they are generally capable of consulting existing expert resources such as books or online encyclopedias, e.g. Wikipedia. As an example, let us consider bird identification. Amateur bird watchers typically rely on field guides to identify observed species. As a general instruction, one has to answer the question “what is most noticeable about this bird?” before skimming through the guide to find the best match to their observation. The answer to this question is typically a detailed description of the bird’s shape, size, plumage colors and patterns. Indeed, in Fig. 1, the non-expert observer might not be able to directly identify a bird as a “Vermillion Flycatcher”, but they *can* simply describe the appearance of the bird: “*this is a bright red bird with black wings and tail and a pointed beak*”. This description can be matched to an expert corpus to obtain the species and other expert-level information.

On the other hand, machines have a much harder time consulting off-the-shelf expert-curated knowledge bases. In particular, most algorithmic solutions are designed to address a *specific* task with datasets constructed *ad-hoc* to serve precisely this purpose. Our goal, instead, is to investigate whether it is possible to re-purpose general image and text understanding capabilities to allow machines to consult already existing *textual* knowledge bases to address a new task, such as recognizing a bird.

We introduce a novel task inspired by the way a layperson would tackle fine-grained recognition from visual input; we name this CLEVER, i.e. Curious Layperson-to-Expert Visual Entity Recognition. Given an image of a subordinate-level object category, the task is to retrieve the relevant document from a large, expertly-curated text corpus; to this end, we only allow non-expert supervision for learning to describe the image. We assume that: (1) the corpus dedicates a separate entry to each category, as is, for example, the case in encyclopedia entries for bird or plant species, etc., (2) there exist no paired data of images and documents or expert labels during training, and (3) to model a layperson’s capabilities, we have access to general image and text understanding tools that do not use expert knowledge, such as image descriptions or language models.

Given this definition, the task classifies as weakly-supervised in the taxonomy of learning problems. We note that there are fundamental differences to related topics, such as image-to-text retrieval and unsupervised image classification. Despite a significant amount of prior work in image-to-text or text-to-image retrieval (Peng et al., 2017; Wang et al., 2017; Zhen et al., 2019; Hu et al., 2019; He et al., 2019), the general assumption is that images and corresponding documents are paired for training a model. In contrast to unsupervised image classification, the difference is that here we are interested in *semantically* labelling images using a secondary modality, instead of grouping similar images (Asano et al., 2020; Caron et al., 2020; Van Gansbeke et al., 2020).

To the best of our knowledge, we are the first to tackle the task of fine-grained image recognition without expert supervision. Since the target corpus is not required during training, the search domain is easily extendable to any number of categories/species—an ideal use case when retrieving documents from dynamic knowledge bases, such as Wikipedia. We provide extensive evaluation of our method and also compare to approaches in cross-modal retrieval, despite using significantly reduced supervision.

## Related Work

In this paper, we address a novel problem (CLEVER). Next we describe in detail how it differs from related problems in the computer vision and natural language processing literature and summarise the differences with respect to how class information is used in Table 1.

**Table 1:** Overview of related topics (K: known, U: unknown)

	Class Information	
Task	Train	Test
FGVR	K	K
ZSL	K	U
GZSL	K	K + U
CLEVER	U	U

### Fine-Grained Recognition

The goal of fine-grained visual recognition (FGVR) is categorising objects at sub-ordinate level, such as species of animals or plants (Wah et al., 2011; Van Horn et al., 2015, 2018; Nilsback and Zisserman, 2008; Kumar et al., 2012). Large-scale annotated datasets require domain experts and are thus difficult to collect. FGVR is more challenging than coarse-level image classification as it involves categories with fewer discriminative cues and fewer labeled samples. To address this problem, supervised methods exploit side information such as part annotations (Zhang et al., 2014), attributes (Vedaldi et al., 2014), natural language descriptions (He and Peng, 2017), noisy web data (Krause et al., 2016; Xu et al., 2016; Gebru et al., 2017) or humans in the loop (Branson et al., 2010; Deng et al., 2015; Cui et al., 2016). Attempts to reduce supervision in FGVR are mostly targeted towards eliminating auxiliary labels, e.g. part annotations (Zheng et al., 2017; Simon and Rodner, 2015; Ge et al., 2019; Huang and Li, 2020). There have also been efforts to classify out-of-domain data by using a semi-supervised approach where in-domain labeled examples alongside unlabeled data are used (Du et al., 2021; Su et al., 2021). In contrast, our goal is fine-grained recognition without access to *categorical* labels during training. Our approach only relies on side information (captions) provided by laymen and is thus unsupervised from the perspective of “expert knowledge”.

### Zero/Few Shot Learning

Zero-shot learning (ZSL) is the task of learning a classifier for unseen classes (Xian et al., 2018). A classifier is generated from a description of an object in a secondary modality, mapping semantic representations to class space in order to recognize said object in images (Socher et al., 2013). Various modalities have been used as auxiliary information: word embeddings (Frome et al., 2013; Xian et al., 2016), hierarchical embeddings (Kampffmeyer et al., 2019), attributes (Farhadi et al., 2009; Akata et al., 2015) or Wikipedia articles (Elhoseiny et al., 2017; Zhu et al., 2018; Elhoseiny et al., 2016; Qiao et al., 2016). Most recent work uses generative models conditioned on class descriptions to synthesize training examples for unseen categories (Long et al., 2017; Kodirov et al., 2017; Felix et al., 2018; Xian et al., 2019; Vyas et al., 2020; Xian et al., 2018), attention-enabled feature extractors (Yu et al., 2018; Zhu et al., 2019; Shermin et al., 2022; Chen et al., 2022). The multi-modal and often fine-grained nature of the standard and generalised (G)ZSL task renders it related to our problem. However, different from the (G)ZSL settings our method uses neither class supervision during training nor image-document pairs as in (Elhoseiny et al., 2017; Zhu et al., 2018; Elhoseiny et al., 2016; Qiao et al., 2016).

### Cross-Modal and Information Retrieval

While information retrieval deals with extracting information from document collections (Manning et al., 2008), cross-modal retrieval aims at retrieving relevant information across various modalities, e.g. image-to-text or vice versa. One of the core problems in information retrieval is ranking documents given some query, with a classical example being Okapi BM25 (Robertson et al., 1995). With the advent of transformers (Vaswani et al., 2017) and BERT (Devlin et al., 2019), state-of-the-art document retrieval is achieved in two-steps; an initial ranking based on keywords followed by computationally intensive BERT-based re-ranking (Nogueira and Cho, 2019; Nogueira et al., 2020; Yilmaz et al., 2019; MacAvaney et al., 2019). In cross-modal retrieval, the common approach is to learn a shared representation space for multiple modalities (Peng et al., 2017; Andrew et al., 2013; Wang and Livescu, 2016; Peng et al., 2016, 2017; Wang et al., 2017; Zhen et al., 2019; Hu et al., 2019; He et al., 2019; Zheng et al., 2021; Wang et al., 2022, 2021). In addition to paired data in various domains, some methods also exploit auxiliary semantic labels; for example, the Wikipedia benchmark (Pereira et al., 2013) provides broad category labels such as *history*, *music*, *sport*, etc.

We depart substantially from the typical assumptions made in this area. Notably, with the exception of He et al. (2019); Wang et al. (2009), this setting has not been explored in fine-grained domains, but generally targets higher-level content association between images and documents. Furthermore, one major difference between our approach and cross-modal retrieval, including (He et al., 2019; Wang et al., 2009), is that we do not assume paired data between the input domain (images) and the target domain (documents). We address the lack of such pairs using an intermediary modality (captions) that allows us to perform retrieval directly in the text domain.

### Natural Language Inference (NLI) and Semantic Textual Similarity (STS)

Also related to our work, in natural language processing, the goal of the NLI task is to recognize textual entailment, i.e. given a pair of sentences (premise and hypothesis), the goal is to label the hypothesis as entailment (true), contradiction (false) or neutral (undetermined) with respect to the premise (Bowman et al., 2015; Williams et al., 2018). STS measures the degree of semantic similarity between two sentences (Agirre et al., 2012, 2013). Both tasks play an important role in semantic search and information retrieval and are currently dominated by the transformer architecture Vaswani et al. (2017); Devlin et al. (2019); Liu et al. (2019); Reimers and Gurevych (2019). Inspired by these tasks, we propose a sentence similarity regime that is domain-specific, paying attention to fine-grained semantics.

## Method

We introduce the problem of layperson-to-expert visual entity recognition (CLEVER), which we address via image-based document retrieval. Formally, we are given a set of images $x_i \in \mathcal {I}$ to be labelled given a corpus of expert documents $D_j \in \mathcal {D}$, where each document corresponds to a fine-grained image category and there exist $K = {\text {|}\mathcal {D}\text {|}}$ categories in total. As a concrete example, $\mathcal {I}$ can be a set of images of various bird species and $\mathcal {D}$ a bird identification corpus constructed from specialized websites (with one article per species). Crucially, the pairing of $x_i$ and $D_j$ is not known, i.e. no expert task supervision is available during training. Therefore, the mapping from images to documents cannot be learned directly but can be discovered through the use of non-expert image descriptions $\mathcal {C}_i$ for image $x_i$.

**Fig. 2:**
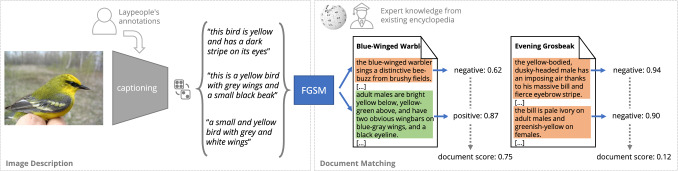
Overview. We train a model for fine-grained sentence matching (FGSM) using layerperson’s annotations, i.e. class-agnostic image descriptions. At test time, we score documents from a relevant corpus and use the top-ranked document to label the image

Our method consists of three distinct parts. First, we learn, using “layperson’s supervision”, an image captioning model that uses simple color, shape and part descriptions. Second, we train a model for Fine-Grained Sentence Matching (FGSM). The FGSM model takes as input a pair of sentences and predicts whether they are descriptions of the same object. Finally, we use the FGSM to score the documents in the expert corpus via voting. As there is one document per class, the species corresponding to the highest-scoring document is returned as the final class prediction for the image. The overall inference process is illustrated in Fig. 2.

### Fine-grained Sentence Matching

The overall goal of our method is to match images to expert documents—however, in absence of paired training data, learning a cross-domain mapping is not possible. On the other hand, describing an image is an easy task for most humans, as it usually does not require domain knowledge. It is therefore possible to leverage image descriptions as an intermediary for learning to map images to an expert corpus.

To that end, the core component of our approach is the FGSM model $f(c_1, c_2) \in \mathbb {R}$ that scores the visual similarity of two descriptions $c_1$ and $c_2$. We propose to train *f* in a manner similar to the textual entailment (NLI) task in natural language processing. The difference to NLI is that the information that needs to be extracted here is fine-grained and domain-specific e.g.  “*a bird with blue wings*” vs. “*this is a uniformly yellow bird*”. Since we do not have annotated sentence pairs for this task, we have to create them synthetically. Instead of the terms entailment and contradiction, here we use positive and negative to emphasize that the goal is to find matches (or mismatches) between image descriptions.

We propose to model *f* as a sentence encoder, performing the semantic comparison of $c_1, c_2$ in embedding space. Despite their widespread success in downstream tasks, most transformer-based language models are notoriously bad at producing semantically meaningful sentence embeddings (Reimers and Gurevych, 2019; Li et al., 2020). We thus follow (Reimers and Gurevych, 2019) in learning an appropriate textual similarity model with a Siamese architecture built on a pre-trained language transformer. This also allows us to leverage the power of large language models while maintaining efficiency by computing an embedding for each input independently and only compare embeddings as a last step. To this end, we compute a similarity score for $c_1$ and $c_2$ as $f(c_1,\,c_2)=h\left( \left[ \phi _1;\,\phi _2;\,\vert \phi _1-\phi _2\vert \right] \right)$, where $[\cdot ]$ denotes concatenation, and *h* and $\phi$ are lightweight MLPs operating on the average-pooled output of a large language model ${{\,\textrm{T}\,}}(\cdot )$ with the shorthand notation $\phi _1 = \phi ({{\,\textrm{T}\,}}(c_1))$.

**Fig. 3:**
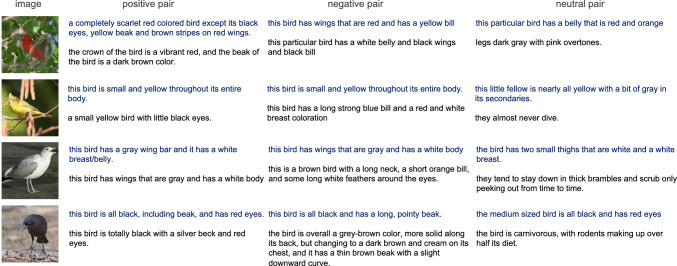
Positive, negative and neutral sentence pairs (CUB-200). We show examples of the automatically generated pairs used to train FGSM. For each pair, the top sentence is a ground-truth caption of the image on the left

#### Training

One requirement is that the FGSM model should be able to identify *fine-grained* similarities between pairs of sentences. This is in contrast to the standard STS and NLI tasks in natural language understanding which determine the relationship (or degree of similarity) of a sentence pair on a *coarser* semantic level. Since our end-goal is visual recognition, we instead train the model to emphasize visual cues and nuanced appearance differences.

Let $\mathcal {C}_i$ be the set of human-annotated descriptions for a given image $x_i$. Positive training pairs are generated by exploiting the fact that, commonly, each image has been described by multiple annotators; for example in CUB-200 (Wah et al., 2011) there are $\vert \mathcal {C}_i\vert = 10$ captions per image. Thus, each pair (from $\mathcal {C}_i \times \mathcal {C}_i$) of descriptions of the same image can be used as a positive pair. The negative counterparts are then sampled from the complement $\bar{\mathcal {C}}_i = \bigcup _{l \ne i}\mathcal {C}_l$, i.e. among the available descriptions for all other images in the dataset. While not perfect, there is a very high chance that these come from images of different classes. We specifically do not add specific rules for constructing negative pairs, other than the fact that they describe different images, as it is not easy to automatically infer reliable noun-attribute combinations from sentences that would allow for further checking (e.g. “the bids is overall yellow, but has dark speckles on its belly”—what color is the belly?) We construct this dataset with an equal number of samples for both classes and train *f* with a binary cross entropy loss.

#### Inference

During inference, the sentence embeddings $\phi$ for each sentence in each document can be precomputed and only *h* needs to be evaluated dynamically given an image and its corresponding captions, as described in the next section. This greatly reduces the memory and time requirements.

### Document Scoring

Although trained from image descriptions alone, the FGSM model can take any sentence as input and, at test time, we use the trained model to score sentences $s\in \mathcal {D}_j$ from an expert corpus against image descriptions $c\in \mathcal {C}_i$. Specifically, we assign a score $z_{ij} \in \mathbb {R}$ to each expert document $D_j$ given a set of descriptions for the *i*-th image: $z_{ij} = \frac{1}{\vert \mathcal {C}_i\times D_j\vert } \sum _{(c, s) \in \mathcal {C}_i\times D_j}{f(c, s)} ,$ Since there are several descriptions in $\mathcal {C}_i$ and sentences in $D_j$, we compute the final document score as an average of individual predictions (scores) of all pairs of descriptions and sentences. Aggregating scores across the whole corpus $\mathcal {D}$, we can then compute the probability $p(D_j \,\vert \, x_i) {\triangleq } \frac{e^{-z_{ij}}}{\sum _{k} e^{-z_{ik}}}$ of a document $D_j \in \mathcal {D}$ given image $x_i$ and assign the document (and consequently class) with the highest probability to the image.

### Bridging the Domain Gap

While training the FGSM model, we have so far only used laypersons’ descriptions, disregarding the expert corpus. However, we can expect the documents to contain significantly more information than visual descriptions. In the case of bird species, encyclopedia entries usually also describe behavior, migration, conservation status, etc. In addition, even the descriptions of visual appearance may utilize specialized jargon. This causes a gap between the style of data observed during training and that encountered during the inference phase. We can adapt the model to the new domain by additionally leveraging information (but not labels) from the target corpus during training. In this section, we thus employ two mechanisms to bridge the gap between the image descriptions and the documents.

#### Neutral Sentences

We introduce a third, neutral class to the classification problem, designed to capture sentences that do not provide relevant (visual) information. We generate neutral training examples by pairing an image description with sentences from the documents (or other descriptions) that do not have any *nouns* in common. Avoiding common nouns in neutral pairs is based on the rationale that if one sentence describes one part (e.g., “black wings”) while another sentence focuses on another (e.g., “white belly”), there is insufficient information to classify the pair as positive or negative. This additionally allows the model to adapt to the style of sentences in the document, which can be very different from image descriptions. Some examples are shown in Fig. 3.

Instead of binary cross entropy, we train the three-class model (positive/neutral/negative) with softmax cross entropy.

#### Score Distribution Prior

Another way of leveraging the document pool during training, without requiring paired data, is by imposing priors on document scoring. To this end, we consider the probability distribution $p(\mathcal {D} \,\vert \, x)$ over the entire corpus $\mathcal {D}$ given an image *x* in a training batch $\mathcal {B}$. We can then derive a regularizer $R(\mathcal {B})$ that operates at batch-level:

1 $$\begin{aligned} {\begin{matrix} R(\mathcal {B}) = \sum _{x \in \mathcal {B}} \Big ( &{} -\langle p(\mathcal {D} \,\vert \, x),\ p(\mathcal {D} \,\vert \, x) \rangle \;\\ &{}+ \sum _{x' \in \mathcal {B} \setminus x } \langle p(\mathcal {D} \,\vert \, x),\ p(\mathcal {D} \,\vert \, x') \rangle \Big ) \end{matrix}} \end{aligned}$$

where $\langle \cdot , \cdot \rangle$ denotes the inner product of two vectors. The intuition of the two terms of the regularizer is as follows. $\langle p(D\,\vert \,x),\, p(D\,\vert \,x) \rangle$ is maximal when the distribution assigns all mass to a single document. Since the score $z_{ij}$ is averaged over all captions of one image, this additionally has the side effect of encouraging all captions of one image to vote for the same document. The second term of $R(\mathcal {B})$ then encourages the distributions of two different images to be orthogonal, favoring the assignment of images uniformly across all documents.

Since $R(\mathcal {B})$ requires evaluation over the whole document corpus for every image, we first pre-train *f*, including the large transformer model *T*, (c.f. Sect. 3.1). After convergence, we extract sentence features for all documents and image descriptions and train only the MLPs $\phi$ and *h* with $\mathcal {L} + \lambda R$, where $\lambda$ balances the 3-class cross entropy loss $\mathcal {L}$ and the regularizer.

## Experiments

We validate our method empirically for bird and plant identification. To the best of our knowledge, we are the first to consider this task, thus in absence of state-of-the-art methods, we ablate the different components of our model and compare to several strong baselines.

### Datasets and Experimental Setup

**Datasets** We evaluate our method on Caltech-UCSD Birds-200-2011 (CUB-200) (Wah et al., 2011) and the Oxford-102 Flowers (FLO) dataset (Nilsback and Zisserman, 2006). For both datasets, Reed et al. (2016) have collected several visual descriptions per image by crowd-sourcing to non-experts on Amazon Mechanical Turk (AMT).

**CUB-200** The Caltech-UCSD Birds-200-2011 (CUB-200) (Wah et al., 2011) contains images of 200 different bird species. The train and test set contains 5,994 and 5,794 images respectively. We have collected expert documents—one document corresponding to each of the 200 categories—by crawling AllAboutBirds^1^ (AAB), which includes bird identification guides made available by the Cornell Lab of Ornithology. Each document consists of an Overview and ID info sections. We obtain basic description from Overview. From page ID info we use Identification, Size & Shape, Color Pattern, Behavior and Habitat. For Size & Shape key we omit the relative size table. For 17 categories that were not found in AAB, we resorted to Wikipedia articles instead. We queried the article for the bird class using MediaWiki API. We use introduction, description, life history sections and ignore the rest. If the class name appears in the text we replace it with the phrase “a bird". We replace any mention of the classes in corpus with the word ‘a bird’ so that the model is unable to cheat by using expert labels.

**Oxford-102 Flowers** The Oxford-102 Flowers (FLO) dataset (Nilsback and Zisserman, 2006) contains images of 102 categories of flowers. We use the official train and test set of 1,020 and 6,149 images respectively. Similar to CUB-200, we create an expert document corpus with one document per category by parsing Wikipedia data using the MediaWiki API. We use summary, cultivation, distribution, description, ecology, flowers, habitat sections and ignore the rest. We replace the expert labels in the corpus with the phrase ‘a flower’.

**Setup** We use the image-caption pairs to train two image captioning models: “Show, Attend and Tell” (SAT) (Xu et al., 2015) and AoANet (Huang et al., 2019). Unless otherwise specified, we report the performance of our model based on their ensemble, i.e. combining captions from both models. As the backbone *T* of our sentence transformer model, we use RoBERTa-large (Liu et al., 2019) fine-tuned on NLI and STS datasets using the setup of (Reimers and Gurevych, 2019).

#### Image Captioning

We consider the following captioning models.

**SAT** We train Show-Attend-and-Tell (SAT) (Xu et al., 2015) for 100 epochs with 64 batch size using the implementation of (Vedantam et al., 2017). We use a ResNet-34 (He et al., 2015) based encoder, and LSTM decoder with input size of 512 and hidden state size of 1800. We use Adam optimizer with learning rate of 0.002. Dropout rate is 0.5, vocabulary size is 5726.

**AoANet** For AoANet (Huang et al., 2019), we extract the bottom-up features with a Faster-RCNN (Ren et al., 2016) backbone pretrained on ImageNet (http://image-net.org/challenges/LSVRC/2015/results) and Visual Gnome(Krishna et al., 2017). The original 2048 dimensional vectors are projected to D=1024. In the decoder LSTM hidden state size is 1024. The vocabulary size for CUB-200 is 1682 and for FLO it is 1711. We use batch size 10 and train for 30 epochs. We use the Adam (Kingma and Ba, 2015) optimizer with initial learning rate of $2e-4$. We anneal the learning rate by 0.8 every 5 epochs. For our experiments we use the implementation from the authors’ repository^2^. During inference, we apply beam search with a beam size of 10 to sample multiple captions from both methods. We have trained the captioning models on the official data splits, reserving 10% of the images from training split for validation for all experiments except the zero-shot experiments where we follow the zero-shot data split.

**BLIP2** We finetune BLIP2 (Li et al., 2023) 2.7b model starting from COCO captioning weights for 5 epochs with learning rate of $1e-5$, batch size of 256, warmup step of 1000. We set image resolution is set to 364, drop path to 0. We use AdamW optimizer with $\beta =(0.9, 0.999)$ and weight decay of 0.05. Layerwise decay rate is set to 0.95.

**OFA** We train OFA (Wang et al., 2022) separately on CUB-200 and FLO datasets. We use OFA-base and start from the COCO captioning weights. We train the model for 5 epochs with learning rate of $1e-5$, batch size of 32. We use cross-entropy loss with label smoothing of 0.1.

For Table 4, we follow the GZSL split proposed in (Xian et al., 2018), using the trainval set to train the captioning models, with 10% of the images being again kept aside for validation. Therefore, we explicitly avoid using “unseen" categories when training the captioning models.

While general image captioning is known to suffer from low diversity, in our fine-grained setting, this is less problematic because of two reasons. Firstly, the vocabulary used is specific to the domain, e.g. , captions describe specific parts (beak, wings, tail) of birds. Secondly, captions describing similar images, such as images of the *same class*, should indeed exhibit similarity rather than distinctiveness.

#### FGSM implementation details

*T* is a sentence transformer with a RoBERTa-large backbone pretrained on the SNLI (Bowman et al., 2015), Multi-Genre NLI (Williams et al., 2018) and STS (Cer et al., 2017) benchmarks. The pretrained model is obtained from the publicly available repository^3^ of (Reimers and Gurevych, 2019). $\phi$ is implemented as a two layer MLP with intermediate and output dimensions of 256 and 64 respectively and $\tanh$ activation function. For *h* we use a linear layer with output dimension of 2 (for the binary classification task). During the first stage of training, we use a constant learning rate of $0.5 \cdot 10^{-6}$ for *T* and $10^{-5}$ for $\phi$ and *h* respectively; weight decay is set to zero for $\phi$ and *h*. We follow (Reimers and Gurevych, 2019) for rest of the hyper-parameters. During the second stage, we aim to reduce the gap between the data that the model is exposed to for training and the target domain. We add the regularizer *R* and fix *T*, pre-computing all embeddings for computational efficiency. We retrain $\phi$ and *h* from scratch with the Adam optimizer (Kingma and Ba, 2015) and an initial learning rate of $10^{-5}$. For $\phi$ we use a three layer MLP with 256, 64, and 32 output dimensions. For *h* we use a linear layer with an output dimension of 3 to predict positive, negative and neutral sentence pairs, training with a cross-entropy loss and the regularizer with a weight factor $\lambda =10$. The neutral sentence pairs are either a pair of captions from two different images that have no nouns in common, or a pair containing a image caption and a random sentence from the target corpus that have no nouns in common. We sample with equal probability from these two pools. The reasoning behind common nouns is that sentences containing the same nouns could potentially describe the same parts—e.g. head, beak, wings—while adjectives are often used as attributes, e.g. *red* wings, *short* beak. Pairs of sentences without common nouns contain neither entailing nor contradicting information, i.e. they describe different objects/parts, and can be thus safely considered as neutral.

We use three metrics to evaluate the performance on the benchmark datasets. We compute top-1 and top-5 per-class retrieval accuracy and report the overall average. Additionally, we compute the mean rank (MR) of the target document for each class. Here, retrieval accuracy is identical to classification accuracy, since there is only a single relevant article per category.

**Table 2:** Comparison to baselines

	CUB-200			FLO		
Method	top-1$\uparrow$	top-5$\uparrow$	MR$\downarrow$	top-1$\uparrow$	top-5$\uparrow$	MR$\downarrow$
ResNet50 (He et al. 2015) (class-supervised)	68.6	90.9	2.6	87.7	97.8	1.3
Random guess	0.5	2.5	100.0	0.9	4.9	51.0
SRoBERTa-STSb (Reimers and Gurevych 2019) (no-ft)	1.3	6.4	73.4	1.1	7.7	45.2
SRoBERTa-NLI (Liu et al. 2019) (no-ft)	1.9	5.3	81.3	0.9	5.7	48.2
Okapi BM25 (Robertson et al. 1995)	1.0	7.5	78.2	1.6	8.0	43.9
TF-IDF (Jones 1972)	2.2	9.7	72.1	1.4	5.0	45.2
RoBERTa (Liu et al. 2019)	4.3	16.6	44.6	1.1	9.6	42.6
Ours	7.9	28.6	31.9	6.2	14.2	39.7

We report the retrieval performance of our method on CUB-200 and Oxford-102 Flowers (FLO) and compare to various strong baselines

### Baseline Comparisons

Since this work is the first to explore the mapping of images to expert documents without expert supervision, we compare our method to several strong baselines (Table 2).

Our FGSM performs text-based retrieval, we evaluate current text retrieval systems.

**TF-IDF** Term frequency-inverse document frequency (TF-IDF) is widely used for unsupervised document retrieval (Jones, 1972). For each image, we use the predicted captions as queries and use the TF-IDF textual representation for document ranking instead of our model. We empirically found the cosine distance and *n*-grams with $n={2,3}$ to perform best for TF-IDF.

**BM25** Similar to TF-IDF, BM25 (Robertson et al., 1995) is another common measure for document ranking based on *n*-gram frequencies. We use the BM25 Okapi implementation from the python package rank-bm25 with default settings.

**RoBERTa** One advantage of processing caption-sentence pairs with a Siamese architecture, such as SBERT/SRoBERTa (Reimers and Gurevych, 2019), is the reduced complexity. Nonetheless, we have trained a transformer baseline for text classification, using the same backbone (Liu et al., 2019), concatenating each sentence pair with a SEP token and training as a binary classification problem. We apply this model to score documents, instead of FGSM, aggregating scores at sentence-level.

**SRoBERTa-NLI/STSb** Finally, to evaluate the importance of learning *fine-grained* sentence similarities, we also measure the performance of the same model trained only on the NLI and STSb benchmarks (Reimers and Gurevych, 2019), without further fine-tuning.

Following (Reimers and Gurevych, 2019) we rank documents based on the cosine similarity between the caption and sentence embeddings.

Our method outperforms all bag-of-words and learned baselines. Approaches such as TF-IDF and BM25 are very efficient, albeit less performant than learned models. Notably, the closest in performance to our model is the transformer baseline (RoBERTa), which comes at a large computational cost (347 sec vs. 0.55 sec for our model per image on CUB-200).

**Class Supervised** For completeness we also report the performance of a class-supervised model in Table 2. Specifically, we train a ResNet50 (He et al., 2016) classifier to predict the class label given an image. We fine-tune the model on each dataset (starting from ImageNet-pretrained weights) for 100 epochs with a learning rate of $1e-4$ and SGD optimizer.

### Ablation & User Interaction

We ablate the different components of our approach in Table 3. We first investigate the use of a different scoring mechanism, i.e. the cosine similarity between the embeddings of *c* and *s* as in (Reimers and Gurevych, 2019); we found this to perform worse (FGSM + cosine).

Next, we evaluate the performance of our model after the final training phase, with the proposed regularizer and the inclusion of neutral pairs (Sect. 3.3). $R(\mathcal {B})$ imposes prior knowledge about the expected class distribution over the dataset and thus stabilizes the training, resulting in improved performance ([2-cls]). Further, through the regularizer and neutral sentences ([3-cls]), FGSM is exposed to the target corpus during training, which helps reduce the domain shift during inference compared to training on image descriptions alone (FGSM w/ ensemble).

Finally, our method enables user interaction, i.e. allowing a user to directly enter own descriptions, replacing the automatic description model. In Table 3 we have simulated this by evaluating with ground-truth instead of predicted descriptions. Naturally, we find that human descriptions indeed perform better, though the performance gap is small. We attribute this gap to a much higher diversity in the human annotations. Current image captioning models still have diversity issues, which also explains why our ensemble variant improves the results.

**Table 3:** Ablations and user study

Method	top-1$\uparrow$	top-5$\uparrow$	MR$\downarrow$
user interaction	11.9	37.5	24.8
FGSM + cosine	4.5	17.8	35.5
FGSM $+\; R(\mathcal {B})$ [2-cls]	7.4	24.6	**29.9**
FGSM $+\; R(\mathcal {B})$ [3-cls]	**7.9**	**28.6**	31.9

On CUB-200 we evaluate scoring functions, captioning models and the regularizer $R(\mathcal {B})$

Bold indicates the best performance

To measure the influence of captions, in Table 5 we evaluate four captioning methods, SAT (Xu et al., 2015), AoANet (Huang et al., 2019), OFA (Wang et al., 2022) and BLIP2 (Li et al., 2023), and show our model’s performance. For this experiment, we train and compare all models without the regularizer $R(\mathcal {B})$. We observe that captioning models that score higher in captioning metrics, e.g., ROUGE, CIDER, etc., also perform well with FGSM. We show examples of captions predicted by the models in Fig. 5

We also an ensemble of captions obtained by two methods, SAT and AoANet. The ensemble is created using the combination of captions of both models and computing the average matching score over all captions. As in almost all tasks, the ensemble improves the performance. The gain, however, is small as (1) captions produced by different models tend to describe similar aspects of the image (Fig. 5), and (2) inaccurate captions will still affect performance, when averaging scores across captions.

**Table 4:** Comparison to cross-media retrieval

Method	sup.	top-1$\uparrow$	top-5$\uparrow$	MR$\downarrow$
Random guess	✗	2.0	10.0	25.0
ViLBERT (Lu et al. 2019)	✗	3.5	14.8	20.2
TF-IDF (Jones 1972)	✗	7.2	28.6	18.9
CLIP (Radford et al. 2021)	✓	10.0	32.9	14.0
DSCMR (Zhen et al. 2019)	✓	13.5	34.7	15.2
Ours	✗	**20.9**	**50.7**	**9.6**

We evaluate the performance of methods on the ZSL split of CUB-200. Our method performs favorably against existing approaches trained with more supervision (sup.=supervision)

Bold indicates the best performance

**Table 5:** Captioning models

Cap. Model	Data.	CLEVER			Image Captioning						
		top1$\uparrow$	top5$\uparrow$	MR$\downarrow$	B-1	B-2	B-3	B-4	M	R	C
SAT (Xu et al. 2015)	CUB	4.3	15.0	42.9	87.0	71.7	57.5	45.5	31.3	62.2	40.7
AoANet (Huang et al., 2019)	CUB	5.7	20.8	38.3	91.3	82.7	**73.9**	**65.4**	38.8	73.8	**75.9**
OFA (Wang et al., 2022)	CUB	4.3	19.8	36.7	92.4	81.8	71.4	62.3	38.0	72.9	70.2
BLIP2 (Li et al., 2023)	CUB	**5.0**	**23.2**	**33.3**	**93.6**	**82.9**	72.4	63.3	**39.5**	**73.8**	75.5
SAT+AoANet	CUB	**5.9**	20.0	36.1	–	–	–	–	–	–	–
BLIP2+AoANet	CUB	5.6	**23.1**	**31.3**	–	–	–	–	–	–	–
SAT (Xu et al., 2015)	FLO	2.8	14.5	39.4	87.1	75.2	65.4	57.7	37.0	69.4	42.6
AoANet (Huang et al., 2019)	FLO	2.7	13.7	39.0	92.0	85.8	78.8	73.5	42.4	79.9	60.6
OFA (Wang et al., 2022)	FLO	2.7	14.5	**38.4**	94.3	88.5	82.6	77.3	45.2	81.3	76.5
BLIP2 (Li et al., 2023)	FLO	**3.0**	**15.4**	38.7	**96.1**	**90.6**	**85.0**	**79.8**	**49.3**	**85.0**	**91.9**
SAT+AoANet	FLO	**3.0**	14.3	**38.2**	–	–	–	–	–	–	–
BLIP2+AoANet	FLO	2.8	**15.4**	**38.2**	–	–	–	–	–	–	–

Performance on the CLEVER task generally increases with captioning performance. (Data.: Dataset, B: BLEU, M: METEOR, R: ROUGE, C: CIDEr-D)

Bold indicates the best performance

**Fig. 4:**
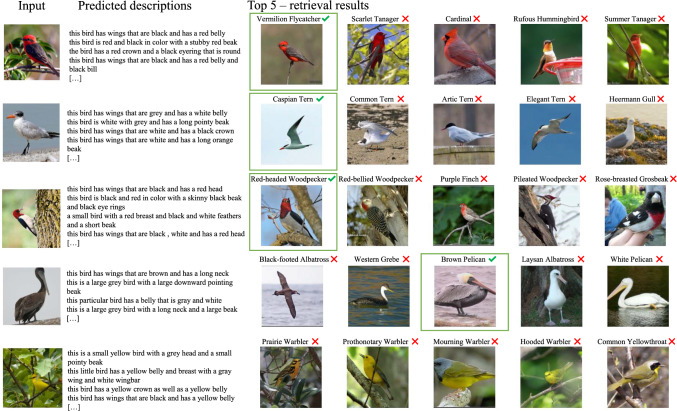
Qualitative Results (CUB-200). We show examples of input images and their predicted captions, followed by the top-5 retrieved documents (classes). For illustration purposes, we show a random image for each document; the image is not used for matching

**Fig. 5:**
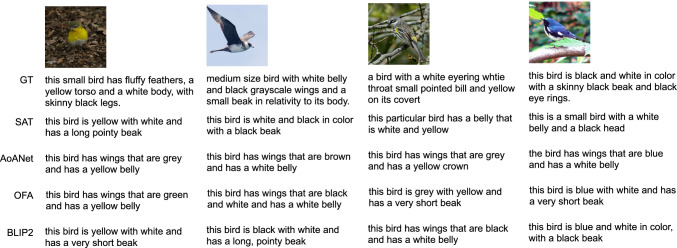
Predictions of Captioning models (CUB-200). We show examples of captions predicted by the captioning models we use - SAT (Xu et al., 2015), AoANet (Huang et al., 2019), OFA (Wang et al., 2022) and BLIP2 (Li et al., 2023)

### Comparison with Cross-Modal Retrieval

Since the nature of the problem presented here is in fact cross-modal, we adapt a representative method, DSCMR (Zhen et al., 2019), to our data to compare to the state of the art in cross-media retrieval. We note that such an approach requires image-document pairs as training samples, thus using more supervision than our method. Instead of using image descriptions as an intermediary for retrieval, DSCMR thus performs retrieval monolithically, mapping the modalities in a shared representation space. We argue that, although this is the go-to approach in broader category domains, it may be sub-optimal in the context of fine-grained categorization.

Since in our setting each category (species) is represented by a single article, in the scenario that a supervised model sees *all* available categories during training, the cross-modal retrieval problem degenerates to a classification task. Hence, for a meaningful comparison, we train both our model and DSCMR on the CUB-200 splits for ZSL (Xian et al., 2018) to evaluate on 50 *unseen* categories. We report the results in Table 4, including a TF-IDF baseline on the same split. Despite using no image-documents pairs for training, our method still performs significantly better.

Additionally, we compare to representative methods from the vision-and-language representation learning space. ViLBERT (Lu et al., 2019) is a multi-modal transformer model capable of learning joint representations of visual content and natural language. It is pre-trained on 3.3M image-caption pairs with two proxy tasks. We use their multi-modal alignment prediction mechanism to compute the alignment of the sentences in a document to a target image, similar to ViLBERT’s zero-shot experiments. The sentence scores are averaged to get the document alignment score and the document with the maximum score is chosen as the class. Finally, we compare to CLIP (Radford et al., 2021), that learns a multimodal embedding space from 400M image-text pairs. CLIP predicts image and sentence embeddings with separate encoders. For a target image we score each sentence using cosine similarity and average across the document for the final score. CLIP’s training data is not public, but we find that there is a high possibility it does indeed contain expert labels as removing class names from documents hurts its performance.

### Qualitative Results

#### Model Performance

In Fig. 4, we show qualitative retrieval results. The input image is shown on the left followed by the predicted descriptions. We then show the top-5 retrieved documents/classes together with an example image for the reader. Note that the example images are not used for matching, as the FGSM module operates on text only. We find that in most cases, even when the retrieved document does not match the ground truth class, the visual appearance is still similar. This is especially noticeable in families of birds for which discriminating among individual species is considered to be particularly difficult even for humans, e.g. warblers (last row).

#### Sentence Composition

**Fig. 6:**
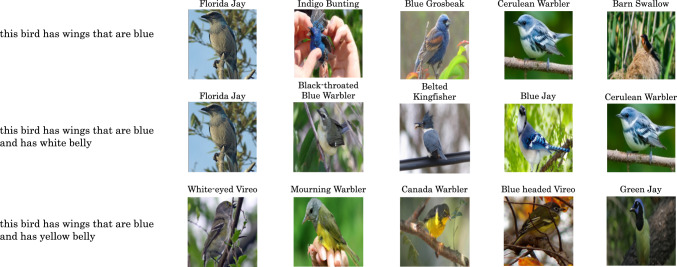
Sentence Composition Results (CUB-200). We show examples of the FGSM model being able to understand compound sentences. We start with a single caption and retrieve the best matching corpus classes in the first row. In the second and third row we add an additional condition to the caption which retrieves even finer-grained classes. For illustration purposes, we show a random image for each document; the image is not used for matching

We observe FGSM, alongside our contrastive learning on captions, also benefits from using a pretrained large language model RoBERTa. We show an example in Fig. 6. The first row shows the retrieval result for the caption "this bird has wings that are blue". As we add another criterion the retrieval becomes more fine-grained, scoring documents with the additional specification more positively.

#### Effectiveness of FGSM Training

**Fig. 7:**
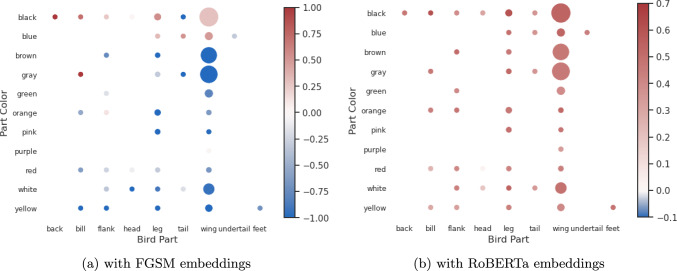
Effectiveness of FGSM training (CUB-200). We use a random caption "this bird has a long wide beak, and black wings, belly, and head." and find its similarity with all the ground truth captions using FGSM and RoBERTa extracted features. The figures show the distribution where the size of the radius denotes the relative occurrence of that pair in captions. We color each color-part pair, e.g., {brown bill, black wings}, using the mean similarity score of all captions containing that pair. Red denotes the mean positive score and blue mean negative score. We find that, as a general-purpose text model, RoBERTa matches all captions with a positive score, while FGSM can contrast based on visual attributes and return positive matches only for colors/parts that are actually present in the caption (Color figure online)

We show the change in fine-grained scoring of sentence-transformer embeddings when training with our method. For this experiment we find the subset of pairs which have one or more color-part pairs present in them (e.g. brown wings, blue tail etc.). For Fig. 7 we randomly sample a caption “this bird has a long wide beak, and blackwings, belly, and head.” and calculated the similarity score for a set of captions using (Fig. 7a) FGSM and (Fig. 7b) RoBERTa. The set is created by combining various part and color names. The figure shows the distribution of similarity scores across the set of color-part combinations. We color the pairs with the mean score of the captions containing that pair. We observe only the captions containing similar colors to black are scored positive by FGSM - showing our model can perform contrastive separation based on visual attributes. Whereas RoBERTa scores all captions positive and cannot discriminate between sentences with different visual attributes. Some other combinations are scored positively by our method, potentially reflecting the expected variance between different human descriptions. For example, blue and black often appear similar in an image depending on lighting and visibility of the part.

**Table 6:** Captioning performance

Dataset	BLEU-1	BLEU-2	BLEU-3	BLEU-4	METEOR	ROUGE	CIDEr-D
CUB-200 (seen)	87.4	72.1	58.0	45.9	31.7	62.6	39.6
CUB-200 (unseen)	86.9	70.9	56.5	44.6	31.1	62.0	38.1
CUB-200 (overall)	87.1	71.4	57.1	45.1	31.4	62.2	38.9
FLO (seen)	88.1	76.3	66.3	58.2	38.6	70.1	54.5
FLO (unseen)	85.6	73.3	63.4	55.1	35.7	67.7	32.6
FLO (overall)	87.0	74.9	65.0	56.8	37.3	69.0	44.5

We verify that the captioning model generalizes to unseen classes

#### Image Description Generalization

As an integral part of our approach, we analyze the performance of the captioning module. In particular, we are interested in the degradation (if any) in the capability of the captioning models to describe images of previously unseen categories. To this end, to understand whether the learned image descriptions are dependent on the training categories, we train the captioning model with the zero-shot learning split and compare the validation performance (in terms of common captioning metrics) between seen and unseen classes in Table 6. We report results using common metrics, BLUE1-4(Cho et al., 2014), METEOR (Denkowski and Lavie, 2014), ROUGE-L (Lin et al., 2004) and CIDEr-D (Vedantam et al., 2015). Interestingly, we find no significant difference in performance between seen and unseen classes, indicating that the model generalizes well to the appearance of novel categories. This is on par with our intuition and motivation for a layperson-inspired system to describe the appearance of objects without necessarily being able to recognize or name them and even when they have never previously encountered a given object.

#### Word Relevance

In Table 7 we show pairs of image descriptions and sentences from the expert corpus, along with the predicted score (after sigmoid). We highlight the importance of individual words which is estimated by masking the word and computing the difference between the new and initial score. The model has learned to pay attention to colors and body parts, which affect its decision the most. The third example also shows that the model is sensitive to negative evidence, as it correctly identifies the color mismatch between the two sentences.

#### Sentence Relevance

While sensitivity to individual words is important, the model also needs to identify which parts of the expert document are relevant, as the descriptions often contain much more information such as the behavior or history of a species. In Tables 8 and 9 we show matching results between a query and a document. We highlight the sentence with the highest matching score within the document, given the query (image description), which indeed identify the visual description within the long document.

**Table 7:**
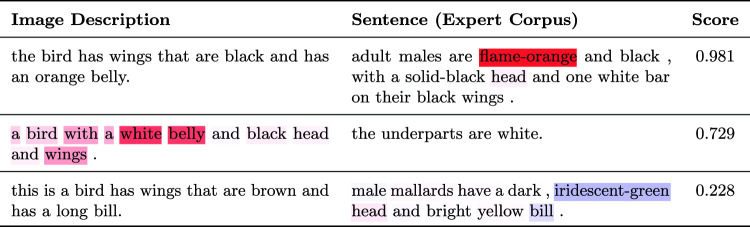
Word relevance visualization

Words are highlighted in blue (red) to highlight positive (negative) changes in the output when the word is occluded (replaced by [UNK]). The darker the shade, the bigger the change in the output

**Table 8:** FGSM qualitative results

Query	This bird has a black body yellow head and gray wings.
Document	With a golden head, a white patch on black wings, and a call that sounds like a rusty farm gate opening, the bird demands your attention. look for them in wesa bird and prairie wetlands, where they nest in reeds directly over the water. they’re just as impressive in winter, when huge flocks seem to roll across farm fields. each bird gleans seeds from the ground, then leapfrogs over its flock mates to the front edge of the ever-advancing troupe. in the midwest and west, look for birds both in freshwater wetlands and in nearby farm fields. though they are striking in appearance, these birds spend a substantial time perched out of view in cattails or reeds, so listen for their harsh check calls and bizarre grinding, buzzing songs in order to pinpoint their location. when searching in farm fields, look for large concentrations of a birds and then scan them carefully. if the bulk of the birds are a bird or some other species, don’t despair-focus on finding a white wing patch or yellow head among the other species. birds are fairly large a birds, with a stout body, a large head, and a long, conical bill. males are striking a birds with yellow heads and chests, and black bodies with prominent white patches at the bend of the wing. females and immatures are brown instead of black, with duller yellow heads. immature males show some white at the bend of the wing, while females don’t. birds breed in loose colonies, and males mate with several females. during the breeding season, they eat insects and aquatic invertebrates. they form huge flocks in winter, often mixing with other species of a birds, and feed on seeds and grains in cultivated fields. birds breed and roost in freshwater wetlands with dense, emergent vegetation such as cattails. they often forage in fields, typically wintering in large, open agricultural areas.
Query	This is a bird with a white belly black back and a red head.
Document	The gorgeous bird is so boldly pata birded it’s been called a “flying checkerboard,” with an entirely crimson head, a snow-white body, and half white, half inky black wings. these birds don’t act quite like most other a birds: they’re adept at catching insects in the air, and they eat lots of acorns and beech nuts, often hiding away extra food in tree crevices for later. this magnificent species has declined severely in the past half-century because of habitat loss and changes to its food supply. look for birds in scattered, open woodlots in agricultural areas, dead timber in swamps, or pine savannas. walk slowly, listening for tapping or drumming, and keep your eyes alert for telltale flashes of black and white as these high-contrast a birds fly in between perches. the red head can be hard to see in strong glare. raucous, harsh weah!. calls will also give away the presence of a bird. birds are medium-sized a birds with fairly large, rounded heads, short, stiff tails, and powerful, spike-like bills. adults have bright-red heads, white underparts, and black backs with large white patches in the wings, making the lower back appear all white when perched. immatures have gray-brown heads, and the white wing patches show rows of black spots near the trailing edge. in addition to catching insects by the normal a bird method of hammering at wood, birds also catch insects in flight and hunt for them on the ground. they also eat considerable amounts of fruit and seeds. their raspy calls are shriller and scratchier than the red-bellied a bird’s. birds live in pine savannahs and other open forests with clear understories. open pine plantations, treerows in agricultural areas, and standing timber in beaver swamps and other wetlands all attract birds. smaller than a northern flicker; about the size of a a bird.

We show several examples of query-document pairs and highlight the best matching sentence

**Table 9:** FGSM qualitative results

Query	This particular bird has a belly that is white with black spots.
Document	The active little bird is a familiar sight at backyard feeders and in parks and woodlots, where it joins flocks of a bird and a bird, barely outsizing them. an often acrobatic forager, this black-and-white a bird is at home on tiny branches or balancing on slender plant galls, sycamore seed balls, and suet feeders. downies and their larger lookalike, the a bird, are one of the first identification challenges that beginning bird watchers master. look for birds in woodlots, residential areas, and city parks. be sure to listen for the characteristic high-pitched pik note and the descending whinny call. in flight, look for a small black and white bird with an undulating flight path. during winter, check mixed-species flocks and don’t overlook birds among the a bird and a bird - birds aren’t much larger than white-breasted a bird. birds are small versions of the classic a bird body plan. they have a straight, chisel-like bill, blocky head, wide shoulders, and straight-backed posture as they lean away from tree limbs and onto their tail feathers. the bill tends to look smaller for the bird’s size than in other a birds. birds give a checkered black-and-white impression. the black upperparts are checked with white on the wings, the head is boldly striped, and the back has a broad white stripe down the center. males have a small red patch on the back of the head. the outer tail feathers are typically white with a few black spots. birds hitch around tree limbs and trunks or drop into tall weeds to feed on galls, moving more acrobatically than larger a birds. their rising-and-falling flight style is distinctive of many a birds. in spring and summer, birds make lots of noise, both with their shrill whinnying call and by drumming on trees. you’ll find birds in open woodlands, particularly among deciduous trees, and brushy or weedy edges. they’re also at home in orchards, city parks, backyards and vacant lots. about two-thirds the size of a a bird between a bird and a bird.
Query	This is a black bird with a white stripe on its face and a red crown.
Document	The bird is one of the biggest, most striking forest birds on the continent. it’s nearly the size of a a bird, black with bold white stripes down the neck and a flaming-red crest. look (and listen) for birds whacking at dead trees and fallen logs in search of their main prey, carpenter ants, leaving unique rectangular holes in the wood. the nest holes these birds make offer crucial shelter to many species including swifts, owls, a birds, bats, and pine martens. look for birds in stands of mature forest with plenty of dead trees and downed logs-deep excavations into rotten wood are telltale signs of this species. also listen for this bird’s deep, loud drumming and shrill, whinnying calls. birds occur at all heights in the forest, and are often seen foraging on logs and near the bases of trees. the bird is a very large a bird with a long neck and a triangular crest that sweeps off the back of the head. the bill is long and chisel-like, about the length of the head. in flight, the wings are broad and the bird can seem a birdlike. birds are mostly black with white stripes on the face and neck and a flaming-red crest. males have a red stripe on the cheek. in flight, the bird reveals extensive white underwings and small white crescents on the upper side, at the bases of the primaries. birds drill distinctive rectangular-shaped holes in rotten wood to get at carpenter ants and other insects. they are loud birds with whinnying calls. they also drum on dead trees in a deep, slow, rolling pata bird, and even the heavy chopping sound of foraging carries well. their flight undulates like other a birds, which helps separate them from a a bird’s straight flight path. birds are forest birds that require large, standing dead trees and downed wood. forests can be evergreen, deciduous, or mixed and are often old, particularly in the west. in the east they live in young forests as well and may even be seen in partially wooded suburbs and backyards. nearly the size of an a bird a bird-sized.

We show several examples of query-document pairs and highlight the best matching sentence

**Table 10:** Contextualization of the CLEVER task

Method	Paired data	#classes annotated	ZSL	GZSL			Sup.
			U$\uparrow$	U$\uparrow$	S$\uparrow$	H$\uparrow$	top-1$\uparrow$
Supervised							
ResNet50 (He et al. 2015)	✓	200	–	–	–	–	68.6
Zero Shot							
LATEM (Xian et al. 2016)	✓	150	49.3	15.2	57.3	24.0	–
ALE (Akata et al. 2015)	✓	150	54.9	23.7	62.8	34.4	–
SAE (Kodirov et al. 2017)	✓	150	61.4	8.8	18.0	11.8	–
Cycle-WGAN (Felix et al. 2018)	✓	150	57.8	46.0	60.3	52.2	–
f-VAEGAN-D2 (Xian et al. 2019)	✓	150	61.0	48.4	60.1	53.6	–
CLEVER							
Ours (ensemble)	✗	0	16.9	6.5	6.7	6.6	7.9

We compare the performance of our method relative to zero-shot learning methods as well as a supervised method. Different from our approach, supervised and (G)ZSL methods utilize expert labels during training. The supervised method uses labels for all 200 classes, while (G)ZSL uses only a subset of these (150). In contrast, our setting uses no expert labels

### Comparison with Zero-Shot Learning

CLEVER is loosely related to the zero-shot learning (ZSL) problem, where, during inference, a model is tasked with classifying samples from classes that have not been observed during training. Unlike CLEVER, however, ZSL explicitly makes use of a *subset* of expert labels during training, and sometimes additional information (attributes, captions, etc.). Consequently, the CLEVER setting uses significantly reduced supervision (i.e., relying only on captions) in contrast to the ZSL setting.

To put our method in context, we compare it against ZSL approaches, even though they employ a higher degree of supervision. Due to the difference in available information during training in ZSL (i.e., some classes are known), it is important to evaluate seen and unseen classes separately. Overcoming this difference is one of the main challenges for *generalized* zero-shot methods (GZSL). In both settings, training is carried out on a set of 150 *seen* classes on CUB-200. In GZSL, during testing, the model has to label an image correctly among all 200 classes, including 50 unseen classes.

In Table 10, we evaluate our method on the ZSL and GZSL splits for CUB-200. To be compatible with the splits used for the (G)ZSL setting, we also train the captioning models and the FGSM module only on the “seen” classes (although no labels are observed). We do not use the regularizer $R(\mathcal {B})$ for this experiment. The lack of expert annotations during training explains the gap in performance between our approach and ZSL/class-supervised methods, as we are tackling a significantly harder problem. However, while many GZSL methods show a large performance gap between seen and unseen classes, our method performs consistently on both sets. This implies that the document pool can be safely expanded to include more classes, if necessary, without the need to re-train for these new classes.

## Discussion

Like with any method that aims to reduce supervision, our method is not perfect. There are multiple avenues where our approach can be further optimized.

First, we observe that models trained for image captioning tend to produce short sentences that lack descriptiveness, focusing on the major features of the object rather than providing detailed fine-grained descriptions of the object’s unique aspects (Fig. 5). We believe there is a scope for improvement if the captioning models could extensively describe each different part and attribute of the object. We have tried to mitigate this issue by using an ensemble of two popular captioning networks. However, using multiple models and sampling multiple descriptions may lead to redundancy. Devising image captioning models that produce descriptive fine-grained image descriptions may provide improved performance on CLEVER task; there is an active area of research (Wang et al., 2020a, b) that is looking into this problem.

Second, the proposed approach to scoring a document given an image uses *all* the sentences in the document classifying them as positive, negative or neutral with respect to each input caption. Given that the information provided by an expert document might be noisy, i.e. not necessarily related to the *visual* domain, it is likely worthwhile to develop a filtering mechanism for relevancy, effectively using only a subset of the sentences for scoring.

Third, in-domain regularization results in a significant performance boost (Table 3), which implies that the CLEVER task is susceptible to the domain gap between laypeople’s descriptions and the expert corpus. Language models such as BERT/RoBERTa partially address this problem already by learning general vocabulary, semantics and grammar during pre-training on large text corpora, enabling generalization to a new corpus without explicit training. However, further research in reducing this domain gap seems worthwhile.

Finally, in the recent time there has been an explosion of work on large multi-modal foundation models that are self-supervised with internet scale datasets. These models have been found to contain strong priors about the world (Radford et al., 2021). Our model is trained on a very small scale dataset compared to that, it would be an interesting avenue to explore how the FGSM will scale with data and how to use the existing foundation models as a prior.

## Conclusion

We have shown that it is possible to address fine-grained image recognition without the use of expert training labels by leveraging existing knowledge bases, such as Wikipedia. This is the first work to tackle this challenging problem, with performance gains over the state of the art on cross-media retrieval, despite their training with image-document pairs. While humans can easily access and retrieve information from such knowledge bases, CLEVER remains a challenging learning problem that merits future research.

## Data Availability

**CUB-2011** The dataset analysed during the current study are available at https://www.vision.caltech.edu/datasets/cub_200_2011/**Oxford-Flowers** The dataset analysed during the current study are available at **WikiPedia** The dataset analysed during the current study are available at https://en.wikipedia.org/**AllAboutBirds** The data that support the findings of this study are available from AllAboutBirds but restrictions apply to the availability of these data, which were used under licence for the current study. Webpage: https://www.allaboutbirds.org/

## Footnotes

1 https://allaboutbirds.com

2 https://github.com/husthuaan/AoANet

3 http://sbert.net/models/
